# Deconstructing the genetic basis of spent sulphite liquor tolerance using deep sequencing of genome-shuffled yeast

**DOI:** 10.1186/s13068-015-0241-z

**Published:** 2015-03-31

**Authors:** Dominic Pinel, David Colatriano, Heng Jiang, Hung Lee, Vincent JJ Martin

**Affiliations:** Department of Biology, Centre for Structural and Functional Genomics, Concordia University, 7141 Sherbrooke Street West, Montréal, Québec H4B 1R6 Canada; School of Environmental Sciences, University of Guelph, Guelph, Ontario N1G 2 W1 Canada; Current address: Energy Biosciences Institute, University of California, Berkeley, Berkeley, CA 94704 USA; Current address: Crabtree Nutrition Laboratories, McGill University Health Center, Montreal, Quebec H3A 1A1 Canada

**Keywords:** Evolutionary engineering, Genome shuffling, Reverse engineering, Complex trait, Tolerance, Yeast

## Abstract

**Background:**

Identifying the genetic basis of complex microbial phenotypes is currently a major barrier to our understanding of multigenic traits and our ability to rationally design biocatalysts with highly specific attributes for the biotechnology industry. Here, we demonstrate that strain evolution by meiotic recombination-based genome shuffling coupled with deep sequencing can be used to deconstruct complex phenotypes and explore the nature of multigenic traits, while providing concrete targets for strain development.

**Results:**

We determined genomic variations found within *Saccharomyces cerevisiae* previously evolved in our laboratory by genome shuffling for tolerance to spent sulphite liquor. The representation of these variations was backtracked through parental mutant pools and cross-referenced with RNA-seq gene expression analysis to elucidate the importance of single mutations and key biological processes that play a role in our trait of interest. Our findings pinpoint novel genes and biological determinants of lignocellulosic hydrolysate inhibitor tolerance in yeast. These include the following: protein homeostasis constituents, including Ubp7p and Art5p, related to ubiquitin-mediated proteolysis; stress response transcriptional repressor, Nrg1p; and NADPH-dependent glutamate dehydrogenase, Gdh1p. Reverse engineering a prominent mutation in ubiquitin-specific protease gene *UBP7* in a laboratory *S. cerevisiae* strain effectively increased spent sulphite liquor tolerance.

**Conclusions:**

This study advances understanding of yeast tolerance mechanisms to inhibitory substrates and biocatalyst design for a biomass-to-biofuel/biochemical industry, while providing insights into the process of mutation accumulation that occurs during genome shuffling.

**Electronic supplementary material:**

The online version of this article (doi:10.1186/s13068-015-0241-z) contains supplementary material, which is available to authorized users.

## Background

Mapping genotype to phenotype for complex traits and using these data for the rational design of biocatalysts is a natural progression in an increasingly sophisticated biotechnology industry. Unfortunately, current technologies do not allow for the rapid creation of industrially relevant microorganisms or the ability to access and understand multigenic phenotypic traits. Traditionally, strain improvement has been based on a repetitive cycle of random mutagenesis and selection to improve the phenotypic traits of industrial microbes [1]. Advanced DNA sequencing technology now allows for rapid sequencing of the genomes of these industrial strains to identify the mutations that confer improved phenotypes. However, in resequencing the genomes of randomly evolved strains, a small number of potentially productive mutations are often accompanied by a background of non-productive mutations [2-4]. Extensive functional characterizations of individual genotypic variations are therefore needed to unravel which mutations are associated with the phenotype of interest. Furthermore, our ability to deconstruct complex, multigenic traits is still limited. Possible solutions to these problems include sequencing pools of independent mutants [5], backcrossing non-productive mutations prior to genome resequencing, or combining intercrossing with pool sequencing to assign quantitative trait loci [6] in order to hone in on productive mutations. Nonetheless, resolving such data into manageable and testable hypotheses can be insurmountably challenging.

**Figure 1:**
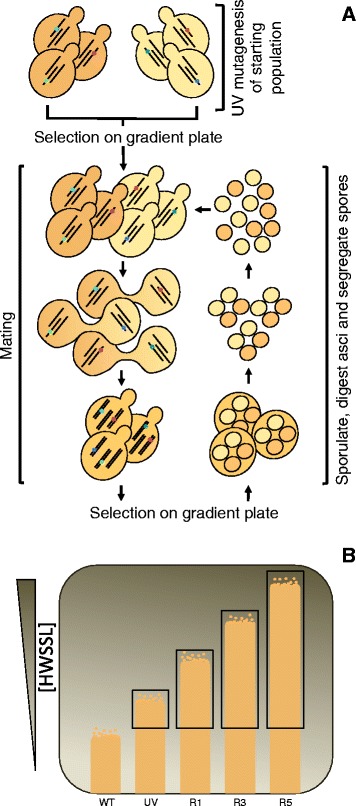
**Meiotic recombination-mediated genome shuffling by recursive breeding for HWSSL tolerance. (A)** A recursive mating methodology was used to create the HWSSL strains and populations used in this study. Large pools of UV mutants and genome-shuffled populations were screened on HWSSL gradient agar plates prior to each round of shuffling. **(B)** Portions of each population that showed more tolerance than the reference (WT) (black boxes) were scraped from gradient plates and used for genome shuffling (different rounds of genome shuffling are depicted - round 1 (R1), round 3 (R3) and round 5 (R5)). Initial UV mutant populations (UV) of each haploid mating type showing enhanced HWSSL tolerance were scraped and used to begin the recursive breeding methodology. Selection on HWSSL gradient plates was carried out between each round of GS in order to enrich the mating pool for strains showing the tolerance phenotype. A portion of each mutant pool (UV through five rounds of GS) was frozen for population sequencing (see ‘Results and discussion’). Individual colonies showing the highest tolerance to HWSSL were isolated from the frontier of growth. HWSSL, hardwood spent sulphite liquor.

In this study, we aimed to mitigate these shortcomings by sequencing a strain created by genome shuffling from a known background strain and tracking mutations throughout the evolving population. This allows for important mutations to be ranked and novel gene targets to be acquired from background mutations. Moreover, genome shuffling (GS) is an alternative to classical strain improvement that is a means to accelerate the evolution of industrial strains in the laboratory and minimize the accumulation of non-productive mutations. The rationale behind GS is to rapidly combine beneficial mutations and cross out deleterious ones, which can be achieved in *Saccharomyces cerevisiae* by recursive pool-wise mating of mutant populations (Figure 1A) [7-10]. This strain engineering technique is particularly powerful to address multigenic, complex phenotypes such as resistance to ethanol, lactic acid, heat and low pH or production of compounds like tylosin or taxol (reviewed in [11]). Theoretically, the background of non-productive or deleterious mutations can be minimized by attenuating mutagen dosage, screening for parental strains that contain productive mutations, followed by trait-enhanced mutant strain recombination to combine mainly productive mutations into a single strain. Furthermore, by its very nature, GS brings interacting mutations together into single strains. Although the utility of GS has been demonstrated repeatedly through phenotypic observation, the nature of the mutations accumulated during the strain evolution has not been tracked through genome resequencing. Sequencing GS isolates, therefore, should yield access to determinants of multigenic traits at single nucleotide resolution, while minimizing non-productive variation discovery. Tracking mutations throughout the population of genome-shuffled strains can then be used to further increase the possibility of finding productive mutations.

Microbial tolerance to lignocellulosic hydrolysates is a complex, multigenic trait that is of significant importance to a biomass-to-fuel/chemical industry. The pretreatment of lignocellulose to fermentable sugars yields many by-products that are inhibitory to fermenting yeasts. The main sources of inhibition come from osmotic pressure, reactive oxygen species (ROS) damage or compounds that include furan aldehydes, primarily furfural and 5-(hydroxymethyl)-2-furaldehyde (HMF), phenolics and organic acids, especially acetic, formic and levulinic acids [12-16]. The biological factors implicated in the tolerance of yeast to lignocellulose fermentation inhibitors have been reviewed [12,13,17]. Ultimately, engineering productive industrial biocatalysts with tolerance traits will be a pervasive biotechnological problem, and rationally engineering these traits will require an understanding of interacting genes and biological processes that affect tolerance. Currently, a lack of knowledge on the multiple cellular processes and genes involved in microbial tolerance to lignocellulosic hydrolysates makes rational engineering of strains resistant to these substrates implausible [8,18,19].

In a previous study [8], we evolved a strain of *S. cerevisiae*, R57, through genome shuffling (Figure 1A,B) that is capable of survival, growth and ethanol productivity in hardwood spent sulphite liquor (HWSSL), a highly inhibitory lignocellulosic substrate generated by the acid bisulphite pulping process [20,21], to levels of tolerance previously unreported. HWSSL contains sugars, lignosulphonates, inhibitory compounds, residual pulping chemicals, ammonia and sulphite [22] and can contain heavy metal ions (iron, chromium, nickel and copper) that originate from the corrosion of pulping and bleaching equipment [23,24]. Some of the major constituents are approximated at the following levels (% *w*/*v*): 0.83% to 1.45% hexose sugars, 1.7% to 2.1% pentose sugars, 0.18% to 0.5% furfural, 0.9% to 1.0% acetic acid, 0.5% to 0.7% sulphate, 1% ammonia and 17% lignosulphonate [8,25]. In evolving R57 through GS, it was hypothesized that beneficial, tolerance-conferring mutations were combined through recursive population-wise meiotic recombination, which yielded a progression towards higher tolerance of the hydrolysate displayed with each subsequent round of GS (Figure 1) [8]. Strain R57 showed improved cross-tolerance to several known inhibitors of lignocellulosic hydrolysates [8], supporting the theory that R57 harbours multiple tolerance-conferring mutations. In this study, we resequenced the genome of R57 in order to discover the mutations that were accumulated to confer hydrolysate tolerance, combined with profiling the relative abundance of R57 mutations in the heterogeneous parental GS populations to probe for the relative phenotypic effect of each discovered mutation and explore mutation recombination in GS-evolved pools.

This study describes a relatively cost-effective way to explore combinatorial space of productive mutations within a single genome. To our knowledge, this is the first study to resequence the genome of a strain evolved through GS and the first resequencing project for a yeast strain specifically evolved for its tolerance to a complex mixture of inhibitors in lignocellulosic substrates.

## Results and discussion

### Genome sequencing of GS-evolved strain R57

The genome of *S. cerevisiae* strain R57 was resequenced in an effort to pinpoint genetic changes associated with its tolerance to HWSSL. Both the parental haploid CEN.PK113-7D and mutant diploid R57 were sequenced and compared at approximately 100-fold and approximately 350-fold coverage per nucleotide (Additional file 1: Data S1), respectively, which allows for meaningful mutation prediction [2]. The relative level of sequence read coverage per chromosome between the strains is similar and suggests the absence of aneuploidy (Additional file 1: Data S1). Insertion/deletion (indel) and copy number variation (CNV) analysis returned no detectable differences between the wild type (WT) and R57 after visual inspection. All of the mutations discovered from the mutation analysis are single nucleotide polymorphisms (SNPs) and were confirmed by Sanger sequencing.

Twenty-one point mutations were found that could affect at least 17 genes, based on location within open reading frames (ORFs) or untranslated regions (UTRs). These include 16 SNPs affecting 12 ORFs, 14 of which lead to missense mutations, with the remaining 2 leading to silent mutations (Table 1). The five mutations not found within ORFs are all located in 5′ or 3′ UTRs. A heterogeneous SNP lies 43 bps 3′ of *BCS1* and is predicted to be part of the 5′ UTR of *YDR374C* [26] and in the 3′ UTR of *WIP1* [27]. This mutation is not included in subsequent analyses due to the ambiguity of the affected gene. Gene ontology categories and interaction maps for the affected genes were generated (Additional file 2: Methods and Additional file 3: Figures S1 and S2), and the mutation analysis results are summarized in Table 1.

**Table 1:** **Point mutations discovered in GS-evolved strain R57**

**Gene**	**Chr**	**Mutation**	**Gene function**	**Genotype**	**SIFT score**
***NRG1***	**IV**	**137C > A (P46Q)**	**Transcriptional repressor, stress tolerance**	**Homo**	**0**
***UBP7*** ^**a**^	**IX**	**2466 T > A (N822K)**	**Ubiquitin-specific protease**	**Homo**	**0.33**
***ART5*** ^**a**^	**VII**	**454C > A (L152I)**	**Regulates endocytosis and turnover of cell-surface proteins by targeted ubiquitination**	**Hetero**	**0.17**
***SSA1*** ^**a**^	**I**	**91C > A (Q31K)**	**ATP-ase, protein folding, heat shock, HSP70**	**Hetero**	**0**
***GDH1*** ^**b**^	**XV**	**47C > T (S16F)**	**Glutamate synthesis from ammonia**	**Hetero**	**0**
***GDH1*** ^**b**^		**68 T > G (F23C)**		**Hetero**	**0**
***ARO1*** ^**b**^	**IV**	**1283C > T (S428F)**	**Catalyzes biosynthesis of chorismate leading to aromatic amino acids**	**Hetero**	**0**
***ARO1*** ^**b**^		**1284C > T (Silent)**		**Hetero**	**-**
***STE5*** ^**b**^	**IV**	**512C > T (S171F)**	**Pheromone-response scaffold protein, forms MAPK cascade complex**	**Hetero**	**0**
***STE5*** ^**b**^		**2649 T > C (Silent)**		**Hetero**	**-**
***MAL11*** ^**b**^	**VII**	**310C > T (P104S)**	**Alpha-glucoside symporter, with high affinity for trehalose**	**Hetero**	**0**
***MAL11*** ^**b**^		**482 T > A (M161K)**		**Hetero**	**0.02**
***GSH1*** ^**c**^	**X**	**T > A (73 bp 5′ UTR)**	**Glutamylcysteine synthetase, glutathione biosynthesis**	**Hetero**	**-**
*PBP1* ^c^	VII	T > C (191 bp 5′ UTR)	Controls mRNA poly(A), stress granule formation and translation control	Hetero	-
*FIT3* ^c^	XV	C > T (42 bp 3′ UTR)	Iron transport	Hetero	-
*NOP58* ^c^	XV	A > T (25 bp 3′ UTR)	Pre-rRNA processing and rRNA synthesis	Hetero	-
YNL058C^d^	XIV	7A > G (K3E)	Unknown function	Hetero	0.42
*DOP1* ^d^	IV	40A > T (N14Y)	Endosome to Golgi transport, ER organization, cell polarity and morphogenesis	Hetero	0.05
*TOF2* ^d^	XI	2141 C > T (S714L)	rDNA silencing, stimulates Cdc14p for mitotic rDNA separation	Hetero	0.27
*SGO1* ^d^	XV	575C > A (S192Y)	Chromosomal segregation and stability	Hetero	0.03

Bold font represents alleles that gain in frequency over GS evolution. ^a^Gene group containing genes that are related to protein homeostasis. ^b^Gene group containing genes bearing more than one mutation in R57. ^c^Gene group containing UTR mutations. ^d^Gene group containing alleles with limited evidence for a phenotypic linkage to HWSSL tolerance.

### Predicting protein function and phenotypic effect for missense mutations in R57

Functional prediction of altered primary protein structure was carried out using the SIFT (Sorting Intolerant From Tolerant) algorithm [28-30] (Table 1). Ten of the 14 missense mutations are expected to affect protein function, with 4 predicted as tolerated by the protein. All but two of the mutations are heterozygous and therefore are expected to have a dominant effect if they contribute to the R57 phenotype. The two homozygous mutations are located within *NRG1* and *UBP7*. Homozygous mutations at these loci suggest that they have been enriched at a high enough population density during GS evolution that they were able to mate with the opposite mating type and may therefore be important to the HWSSL tolerance trait or that spores were insufficiently segregated during GS (Figure 1A), promoting homozygosity.

Several genes contain multiple mutations in R57. Genes bearing more than one mutation in R57 suggest that the affected gene is important to the phenotype and the mutations have accumulated due to GS evolution and screening. Two missense mutations affect both *GDH1* and *MAL11*. Gdh1p bears two mutations in R57, S16F and F23C. Visual inspection of the mapping alignment shows that the two mutations are never located on the same sequence read, and therefore, both versions of *GDH1* are mutated in R57. The close proximity of these two mutations suggests that a mutation in this region of *GDH1* may yield a phenotypic trait that has been selected for through GS evolution. The R57 *MAL11* gene contains two missense mutations (leading to P104S and M161K). Cloning and sequencing of *MAL11* from R57 shows that the two mutations are not located on the same allele. *STE5* and *ARO1* each bear a second silent mutation that may be present due to close genetic linkage to the productive mutation or lead to a non-obvious phenotypic modification, such as altered mRNA stability, and thereby arise in R57 through selection.

### Analysis of mutation loci in GS-evolved heterogeneous populations

**Figure 2:**
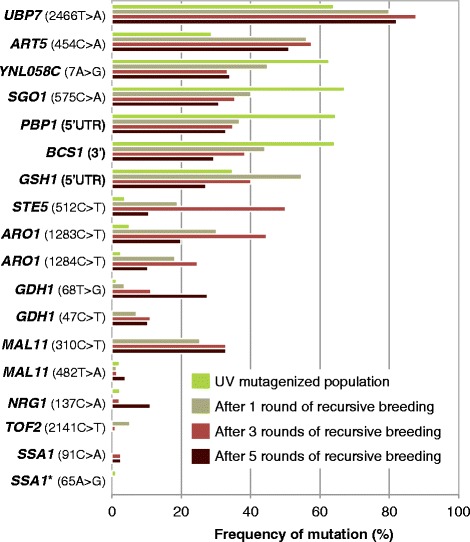
**Frequency of R57 mutations throughout GS evolution.** The haploid UV mutant population (UV), generated from a single round of UV mutagenesis, and rounds 1, 3 and 5 of GS evolution (R1, R3 and R5, respectively) were PCR amplified at the R57 mutation loci and deep sequenced to determine the relative frequency of sequence reads bearing mutation (above a threshold of 1% of total sequence reads). The asterisk denotes mutations discovered that are not found in R57. UV, ultraviolet.

We deeply sequenced the R57 mutation loci within the pooled mutant populations generated during the genome shuffling experiments [8]. The mutation loci were PCR amplified from DNA extracted from the heterogeneous populations and sequenced. There were a total of 3.76 × 10^6^ reads with an average read length of 104 bp and an average fold coverage of 1.35 × 10^5^ reads per nucleotide sequenced. Eighteen SNPs were called (Figure 2). We assessed the frequency of each sequence read that contains a mutation within the total number of sequence reads spanning each locus (Figure 2). These data were used to predict mutations that may have arisen in single parental strains, due to similar prevalence within a population, or may be epistatic, and to probe for changes in SNP frequency between populations that could indicate the relative influence of those alleles on phenotype. Several mutant alleles increase in frequency over GS evolution, and we hypothesize that this increase is due to a beneficial phenotypic effect that is generated through GS evolution and repeated selection. These include the *ART5*, *UBP7*, *SSA1*, *STE5*, *NRG1*, *MAL11* (leading to Mal11p - P104S) and both *GDH1* mutations (Figure 2). This analysis also yields an additional SNP that falls within the area of PCR amplification, affecting *SSA1* 26 bp upstream of the R57 mutation and leading to a D22G missense mutation, which further supports a determinant role for *SSA1* in the observed phenotype. The *NOP58*, *STE5* (silent), *DOP1*, *FIT3* 3′ UTR mutations were not located at a high enough density within the sequenced mutant populations to surpass our detection threshold.

Five mutations (located upstream of *BCS1* and *PBP1* and in the ORFs of YNL058C, *SGO1* and *UBP7*) are represented at approximately 60% frequency in the UV mutant population. Due to the virtually identical representation of these five mutations within the UV mutant population, we hypothesized that they may arise from a single, highly tolerant haploid strain. The mutated loci identified in R57 were sequenced from seven random, discernible colonies selected from the frontier of growth for the UV mutant populations on HWSSL gradient agar plates (Figure 1B). Each of the single colonies contained the *BCS1*, *PBP1*, YNL058C, *SGO1* and *UBP7* mutations, corroborating our hypothesis that a single mutant strain present after UV mutagenesis was likely enriched to represent a large portion of that population. Assuming this mutant strain harboured at least one particularly productive mutation, propagation of these alleles to strain R57 may be a likely outcome. Heterogeneous population sequencing shows that of these five mutations, only the *UBP7* mutation increased in frequency through GS evolution while the other four decreased in frequency (Figure 2). We hypothesize that this finding is indicative of a set of four non-productive or less important mutations found with the productive *UBP7* mutation within a single genome, and when meiotic recombination occurs, linkage to the productive mutation diminishes until it reaches a steady state within the evolving population.

The mutation in the 5′ UTR of *GSH1* was identified in approximately 30% of sequence reads generated for this locus (Figure 2) in the UV population and was also identified in each of the seven UV mutant isolates that contain *BCS1*, *PBP1*, YNL058C, *SGO1* and *UBP7* mutations. It is therefore likely that all six mutations arose in a single mutant strain. The reason for the discrepancy in allele frequency between *GSH1* and the other five mutations from UV mutant population sequencing is unknown but may be a population sequencing artefact. Unlike *BCS1*, *PBP1*, YNL058C and *SGO1*, the 5′ UTR *GSH1* mutation increases in frequency in the first three rounds of evolution (Figure 2) and therefore, as with *UBP7*, more likely contributes to the tolerance phenotype than the other four mutations.

Several mutations that comprise a large part of the first three rounds of GS, and are therefore likely playing a determinant role in HWSSL tolerance, decrease in population frequency in the fifth round of GS (*UBP7*, *ART5*, *ARO1*). We hypothesize this occurs due to competition from strains bearing mutations that were rare in the initial mutant pools (that is, *NRG1* and *GDH1*) or strains harbouring rare recombination events of multiple mutations that have resulted in augmented fitness.

### Analysis of R57 SNPs in isolates of GS round 5 heterogeneous population

**Figure 3:**
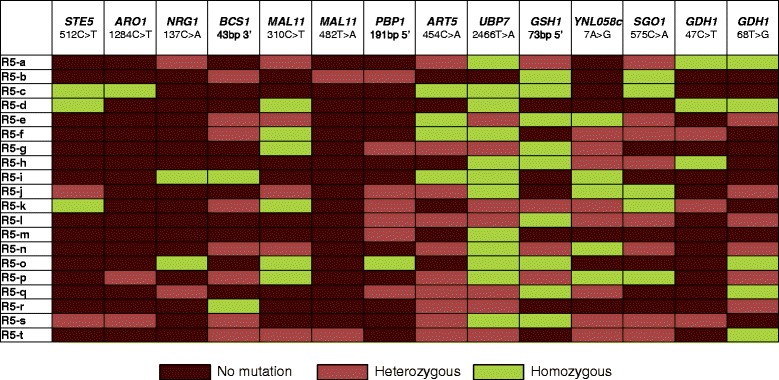
**Prevalent mutation loci found in 20 individual isolates from the fifth round of GS.** Twenty (R5-a-t) strains from the fifth round (R5) of GS were isolated, and the most prevalent mutation loci were PCR amplified and sequenced to determine their presence within each strain. The presence of homozygous mutation (green), heterozygous mutation (red), as determined by a mixed Sanger sequence at the nucleotide of interest, or no mutation (brown) are depicted for each strain.

**Figure 4:**
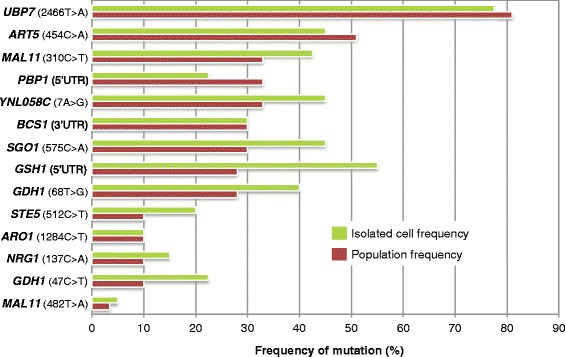
**Comparison of mutation frequencies between population and single colony at mutation loci.** Twenty single colonies were sequenced using the Sanger method at the most prevalent R57 mutation loci for comparison to deep sequencing results of mutation loci within the entire GS-evolved population.

To identify possible combinations or permutations of mutations enriched through evolution, 20 strains isolated from the growth frontier of the fifth round of recursive GS (Figure 1B lane R5) were sequenced via the Sanger method at each of the mutation loci. All of the strains show heterozygosity in at least one of the mutation loci. The results show a heterogeneous population of mutations that are found together in one strain (Figure 3). Of these isolates, ≥70% contain at least one mutation in *UBP7*, *ART5* and either of the *GDH1* mutation loci, which further supports determinant roles for these genes on the phenotype. Several of the strains contain very few of the R57 mutations, which may indicate that not all R57 mutations are needed for HWSSL tolerance and likely that other unidentified mutations present within the round 5 population contribute to the tolerance trait. The percentages of mutated alleles within mutant populations as enumerated by population sequencing and single colony sequencing are similar (Figure 4); discrepancies in these frequencies may be due to the relatively large difference in sample size. General trends as to allele frequency are easily apparent and support our ability to generalize allele frequency within GS mutant populations by sequencing PCR-amplified mutation loci. The GS population sequencing data support determinant roles in HWSSL tolerance for a large portion of the mutated R57 genes based on their pervasiveness throughout the evolving populations. However, the most highly enriched mutations that increase over the strain evolution are of particular interest for reverse engineering studies. These include *UBP7*, *GDH1*, *ART5*, *ARO1*, *STE5* and *MAL11.*

### RNA-seq gene expression analysis

**Figure 5:**
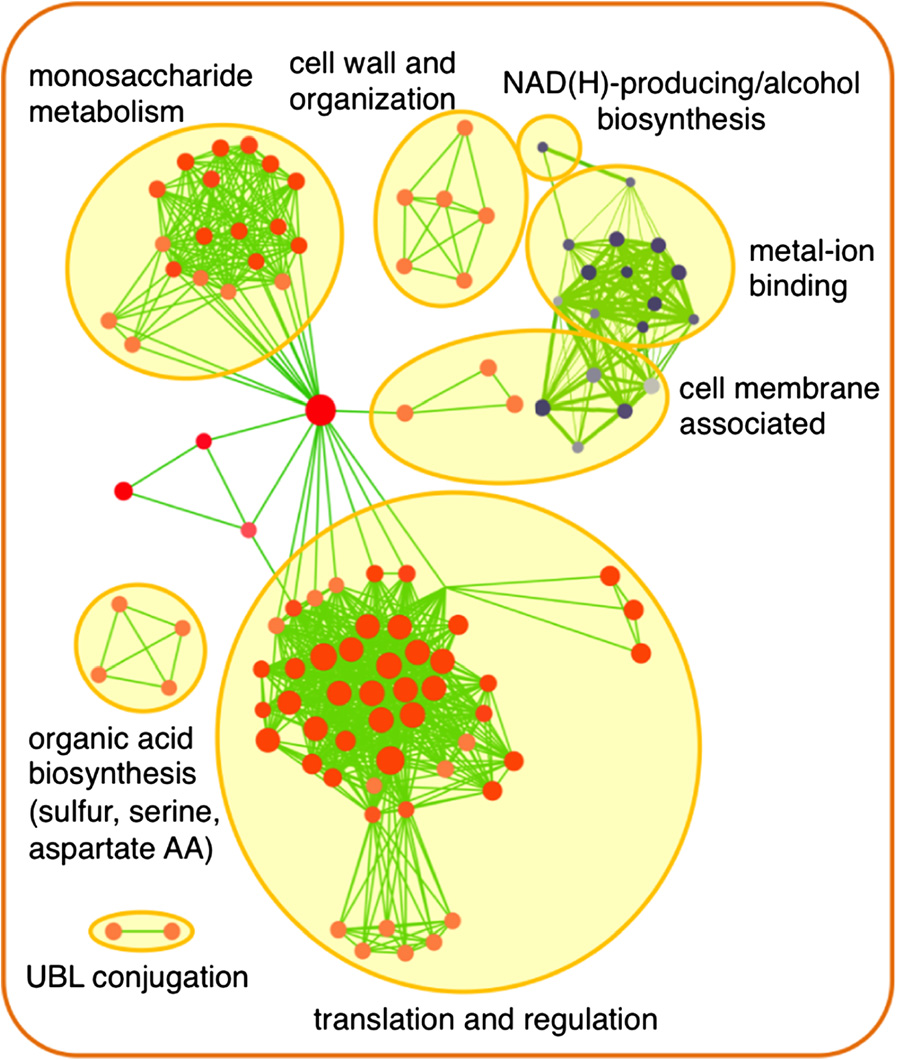
**Enrichment clustering of differentially expressed genes from RNA-seq analysis.** WT and R57 were compared for constitutive differential expression of genes when grown in SD medium. Gene lists were compiled for significantly (*P* < 0.05) upregulated >2-fold or significantly downregulated <2-fold differentially expressed genes. Red colours represent upregulation, while blue colours represent downregulation. Darker shades represent a relatively higher confidence of enrichment score. Larger node sizes represent relatively larger numbers of differentially regulated genes associated with the given ontology category as compared to the full gene. Smaller distance between nodes denotes a higher degree of relationship between ontology categories, while thicker edge lines (green) denote a relatively higher degree of similarity between category nodes in terms of the degree of overlap between the specific gene sets they are associated with. AA, amino acid; UBL, ubiquitin-like.

To measure the impact of the mutations on gene expression in strain R57 and to probe for biological processes related to HWSSL tolerance, the gene transcription profile of strain R57 was compared to the WT diploid under control conditions (growth in defined medium, see ‘Materials and methods’) (Additional file 4: Data S2). Functional clustering was performed on the differentially expressed genes to discover enriched functional roles of gene products and biological pathways of interest (Figure 5).

These analyses identified 149 differentially expressed genes (>2-fold) (Additional file 3: Figure S3 and Additional file 4: Data S2). None of the 16 genes harbouring a mutation (Table 1) are found in this group with the exception of *NRG1*, which is upregulated 3-fold. Clustering of the 131 upregulated R57 genes as compared to the WT includes the major cluster of translation-related genes, mainly associated with ribosome biogenesis and translation regulation and 15 genes related to monosaccharide metabolism. These findings suggest a more active metabolism of R57 in early stationary phase, which may be related to growth differences between the WT and R57 [8]. Indeed, R57 displays a similar growth rate to the WT but reaches a lower optical density (OD) at stationary phase under non-inhibitory conditions with residual glucose remaining in the R57 medium (Additional file 3: Figure S4). The remaining upregulated enrichment clusters include genes related to cell wall organization, the cell membrane, ubiquitin-like (UBL) conjugation and organic acid synthesis pathways. Only 18 genes were downregulated under non-inhibitory conditions, resulting in 3 clusters of genes that are highly enriched (Figure 5). These include genes related to NADH/alcohol metabolism and metal-ion metabolism or are associated with cellular membrane transport or lipid metabolism.

### *UBP7* and the ubiquitin-mediated protein homeostasis machinery are determinants of HWSSL tolerance

*UBP7* bears a mutation that gains in frequency to represent a large portion of the GS-evolved population (Figure 2). Functional analysis suggests highly probable effects on protein structure and function due to this mutation, and *UBP7* is known to have a high degree of interaction with mutated R57 genes (Table 1, Additional file 2: Methods, Additional file 3: Figures S1 and S2). The RNA-seq data also shows enrichment clustering for increased expression of genes that encode proteins related to the ubiquitin-mediated proteolytic machinery (UBL conjugation) (Figure 5). However, the genes within these UBL-conjugated gene clusters are associated with diverse biological processes, many of which do not play a direct role in the ubiquitination of proteins or ubiquitin-mediated proteolysis. Two notable exceptions, *UBI2* and *UBI3*, which show increased expression in R57 relative to the WT (2.7- and 2.8-fold, respectively; Additional file 4: Data S2), encode ubiquitin fused to ribosomal proteins [31] and are responsible for generating ubiquitin as a fusion protein that is then cleaved to yield free ubiquitin by deubiquitinases. Most of the UBL conjugation cluster genes encode proteins that are regulated by this mechanism. Many of these genes are stress-tolerance related (*RHR2*, *PUN1*, *ENA5*, *PDR5*, *PDR12*). This suggests that stress tolerance genes showing increased expression due to HWSSL exposure may be also differentially controlled at the protein level by a modified ubiquitination machinery. Altogether, a significant portion of the HWSSL tolerance trait shown by R57 seems to be a direct result of changes in ubiquitin-mediated proteolytic pathways.

Protein damage and aggregation are likely a source of toxicity in cells exposed to lignocellulosic hydrolysates and have at least been partially shown to arise due to ROS damage from furan aldehyde exposure [32]. Cells regulate protein quality through destruction of misfolded or damaged polypeptides largely through selective, energy-dependent labelling with ubiquitin leading to digestion by the 26S proteasome complex [33]. *UBP7* encodes a ubiquitin-specific protease that cleaves ubiquitin-protein fusions [34], and as such, it is part of this ubiquitin-induced signalling machinery of the cell [35-37]. The cell’s requirement for available ubiquitin increases during stress exposure [38]. Deubiquitinating enzymes act to recover ubiquitin from ubiquitin-protein conjugates and may therefore have a direct bearing on cellular protein and ubiquitin homeostasis [37]. It has already been shown that mutations within a deubiquitinase enzyme, *UBP6*, can dramatically change steady-state ubiquitin levels within a cell [39], which is known to affect tolerance to a variety of stressors [40-42] and yeast prion toxicity [42]. Furthermore, upregulation of *UBP13*, another yeast deubiquitinating enzyme, is beneficial to cells under cold stress and suggests that altering ubiquitin-induced signalling may be a viable path towards other forms of stress tolerance [43].

**Figure 6:**
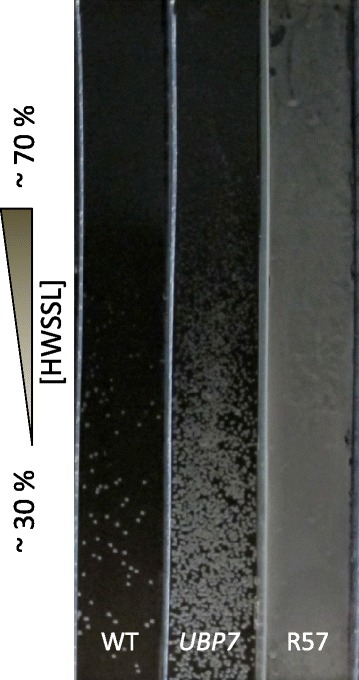
**Testing of** ***UBP7*** **mutation using HWSSL gradient agar plate screening.** A HWSSL gradient agar plate (approximately 30% to 70% HWSSL from bottom to top of plate) was spread with cells from cultures of (from left to right lanes) CEN.PK 113-7D diploid (WT), CEN.PK 113-7D diploid bearing the homozygous *UBP7* mutation from R57 (*UBP7*), and strain R57 (R57). HWSSL, hardwood spent sulphite liquor; WT, wild-type.

In order to test the role of the *UBP7* 2466 T > A mutation in hydrolysate tolerance, we replaced both WT copies of *UBP7* with this gene variant in a diploid WT CEN.PK background. The homozygous *UBP7* 2466 T > A strain was able to colonize a higher concentration of HWSSL on gradient agar plate screening (Figure 6) compared to the WT, but its tolerance to HWSSL is still below that of R57. The phenotype conferred by the *UBP7* mutant does not reconstitute the full HWSSL tolerance displayed by R57 and supports the hypothesis that the high level of tolerance shown by R57 is a result of several mutations incorporated through GS.

One role of ubiquitination is the internalization of cell surface proteins [44-46]. This function relates to Art5p, which belongs to the ART (arrestin-related trafficking) family of proteins that are believed to function as adaptors for Rsp5p, a ubiquitin ligase that promotes endocytosis of plasma membrane proteins, including transporters, targeting damaged or unneeded plasma membrane proteins for vacuolar degradation [47]. Mutation of *ART5* may represent a way for R57 to regulate destruction of proteins damaged by HWSSL stress or direct changes to the plasma membrane in order to respond more efficiently to the toxic HWSSL environment. As might be expected of a leucine to isoleucine mutation, like that found in the R57 Art5p L152I protein, this change is expected to be tolerated by Art5p (Table 1). Nevertheless, the high and increasing frequency of the *ART5* 454C > A mutation shown in the mutant pool sequencing experiment (Figure 2), the differential regulation of cell surface remodelling genes between the WT and R57 (Additional file 4: Data S2) and the proven role of ubiquitin-mediated degradation-machinery gene *UBP7* in HWSSL tolerance suggest that this mutation might also play a determinant role in HWSSL tolerance.

### Nrg1p as a determinant in the inhibitor tolerance trait

As the sole transcription factor-encoding gene located amongst R57 mutations, the *NRG1* 137C > A may result in the most pervasive phenotypic consequences. The homozygous P46Q mutation of Nrg1p was predicted to be non-tolerated with a SIFT score of 0.00 (Table 1), as proline is strictly conserved at this position and its substitution would result in altered protein function. SNP analysis of the GS-evolved populations showed a diminished prevalence of this mutation after mating of the UV-treated haploid population (Figure 2), leading to the hypothesis that the *NRG1* mutation results in a recessive, loss-of-function mutation. The *NRG1* 137C > A allele gains prevalence in the GS populations after that point, present at a frequency of approximately 2% after three rounds and approximately 11% after five rounds of pool-wise recursive breeding, when two copies of the allele are more likely present in single strains.

Nrg1p recruits the Tup1p-Cyc8p complex to repress gene expression. Therefore, the Nrg1p P46Q mutation may decrease repression of Nrg1p-controlled genes. *NRG1*, which self-regulates its transcription [48-51], was upregulated 3-fold in R57 over WT (Additional file 4: Data S2). The closely related transcription factor *NRG2* was similarly upregulated approximately 5-fold in R57 over WT. In addition, four of the five most highly upregulated genes in R57 are known to be regulated by Nrg1p. These genes include *CWP1* (approximately 13-fold upregulated), YLR015C (approximately 44-fold upregulated), *GAT3* (approximately 23-fold upregulated) and *TDA6* (approximately 14-fold upregulated) (Additional file 4: Data S2). In *S. cerevisiae*, the most significant transcriptional responses governed by Nrg1p are all related to a multitude of stress conditions [52]. One of the main functional gene categories controlled by Nrg1p is related to peroxide tolerance, which is a specific trait of R57 [8]. Additionally, Vyas *et al*. reported that Nrg1-2p regulates a set of stress response genes and *Δnrg1Δnrg2* deletion mutants exhibit tolerance to oxidative stress and salt exposure [53], a trait that is also shared by R57 [8].

R57 also displays acetic acid tolerance after pre-exposure to HWSSL [8]. Nrg1p can directly repress genes that are activated by the downstream action of protein kinase Snf1p [54], which stimulates upregulation of stress responsive genes [55] and the transcription activator Haa1p, which imparts acetic acid tolerance [56]. Many of the genes highlighted as members of enriched clusters that show increased expression in R57 are known constituents of the Haa1p regulon including *AQR1*, *HSP26*, *MSN4*, *PDR12*, *PDR16*, *SPI1*, *SUR2*, *SSE2*, *TDA6*, *TPO1*, *TPO2* and *TPO3* (Additional file 4: Data S2). Genes like Haa1p-dependent *TPO2* and *TPO3* (Nrg1p-regulated [49]) show a prominent effect on acetic acid tolerance [57]. Overall, our data support a strong role for Nrg1p in the control of hydrolysate inhibitor tolerance.

### A determinant role for *GDH1* in HWSSL tolerance

The presence of mutations predicted as non-tolerated in both copies of *GDH1* and their close proximity (Table 1) strongly suggest a determinant role for *GDH1* in HWSSL tolerance. Furthermore, population sequencing shows a steady increase in mutant allele frequency at both loci (Figure 2). *GDH1* encodes an NAD(P)H-dependent glutamate dehydrogenase that catalyses the reductive amination of α-ketoglutarate to yield glutamate, responsible for the majority of cellular nitrogen in *S. cerevisiae* via ammonium assimilation [58]. *GDH1* is recognized as a determinant of resistance to acetic acid [59,60]. Likewise, recent proteomics studies using a strain that is tolerant of furfural, phenol and acetic acid show a downregulated nitrogen assimilation machinery, including Gdh1p [59]. It is believed this occurs in order to slow growth and allow stress tolerance mechanisms to protect the cell more effectively. A decrease in biomass yield exhibited by R57 relative to the WT under permissive conditions (Additional file 3: Figure S4) suggests that metabolism has shifted to a state of decreased resource utilization efficiency. As *GDH1* is a central hub of nitrogen metabolism, microbial substrate tolerance engineering studies that focus on this gene are warranted, especially on the HWSSL substrate that was used to evolve strain R57, which is generally high in ammonia content (approximately 1% *w*/*v* [22]) and could lead to ammonia toxicity [61]. Bayer *et al.* recently showed that by increasing expression variability of *GDH1* alone, one can tune the metabolism of a cell so that it responds more efficiently to limiting or toxic levels of ammonia [62].

The NADPH cofactor requirement of *GDH1* is also a major consideration in attempting to explain the consequences of the *GDH1* mutations. Yeast detoxifies the furan aldehyde inhibitors found in lignocellulosic hydrolysates by way of NADH/NADPH requiring enzymes [13]. Differential gene expression analysis between the WT and R57 show increased expression of *GRE3* (Additional file 4: Data S2) in R57, encoding for a methylglyoxal reductase that can reduce furan aldehyde inhibitors via NADH [14]. The NAD(P)H cofactor usage of R57 may be modulated by a modified GDH1p, providing the reducing equivalents needed to detoxify the HWSSL furan aldehyde inhibitors.

### Sequencing supports determinant HWSSL tolerance roles for mutated genes *SSA1*, *ARO1*, *MAL11* and *GSH1*

Given the GS methodology, it is likely that several of the accumulated mutations influence hydrolysate tolerance. This study was able to generate more restricted evidence that the *SSA1*, *ARO1*, *MAL11* and *GSH1* mutations found in R57 may be affecting HWSSL tolerance.

*SSA1*, also related to protein homeostasis, bears a mutation in R57. Ssa1p is a member of the heat shock 70 (Hsp70) family of proteins, which consists of highly conserved, broad specificity, essential protein chaperones (for reviews, see [63,64]). The tendency of harsh conditions to damage proteins and lead to aggregation [33] likely makes the role of Hsp-encoding genes important for HWSSL tolerance. The R57 Q31K mutation of Ssa1p is located at a highly conserved residue, as predicted by SIFT (Table 1). Although not highly represented in population sequencing, the discovery of an adjacent *SSA1* mutation (D22G) that is also present within the tolerant population (Figure 2) suggests that this region of Ssa1p may have bearing on the R57 phenotype. The structure of Ssa1p contains two distinct domains, the nucleotide-binding domain (NBD) that is responsible for binding and hydrolyzing ATP and the substrate-binding domain which can bind short hydrophobic segments of incompletely folded or unfolded polypeptides, in order to prevent adverse aggregation [63]. The Q31K mutation of Ssa1p is located in the NBD, and when the *Escherichia coli* Hsp70 homolog DnaK is used as a structural reference, the mutation is shown to lie adjacent to the ATP-binding pocket [65]. Although the Q31K and D22G mutations in Ssa1p have not been studied thus far, residues in this area of the protein have been shown to influence its NBD function and folding activity [65,66].

Furthermore, Aro1p, which catalyses steps 2 to 6 of the chorismate pathway leading to synthesis of aromatic amino acids [67], harbours a predicted phenotype-conferring mutation that gains in frequency within sequenced mutant pools. After an acetic acid challenge, aromatic amino acid synthesis and tryptophan synthesis in particular are pathways that are found to be upregulated [60], while mutants auxotrophic for aromatic amino acid synthesis show acetic acid sensitivity [68] and deletion of *ARO1* leads to sensitivity to osmotic and ethanol stress [69].

Mal11p bears two mutations that pervade and increase in the GS-evolved HWSSL-tolerant population. Mal11p is a trehalose-H^+^ symporter [70,71] and could be related to osmotic stress protection via trehalose transport or pH stasis due to its proton requirement. Although the effect of *MAL11* on tolerance to industrial processes has not been demonstrated, it is a common trend for the genome of industrial yeast strains to show a loss or reduction of *MAL11* genes [72-74].

Finally, the mutation in the 5′ UTR of *GSH1* may potentially be affecting redox homeostasis by influencing glutathione levels; *GSH1* can influence tolerance to lignocellulosic hydrolysate inhibitors in this way [75]. Glutathione is comprised of glycine, cysteine and glutamate and is a major redox buffer of the cell, cycling between its reduced and oxidized form, relying on NADP(H) for recycling. Hydrogen peroxide induces *GSH1* transcription but relies on the presence of intercellular amino acid pools, namely glutamate, glutamine and lysine to induce glutathione production [76]. Therefore, modified glutamate assimilation via *GDH1* mutation and the 5′ UTR mutation in *GSH1* both potentially affect intercellular glutamate pools and concomitant expression of *GSH1*. Between the WT and R57, genes that lead to cysteine biosynthesis (*HOM2*, *MET16*, *MET17* and *MET3*), along with genes leading to glutamate and lysine biosynthesis (*HOM3*, *LYS9* and *LYS12*), are upregulated (Additional file 4: Data S2). This finding suggests that R57 has upregulated pathways towards glutathione precursor generation as part of its physiology and constitutes a possible link to the *GSH1* 5′ UTR mutation.

### GS population sequencing provides evidence against prominent phenotypic roles of isolated mutations

Although some of the remaining R57 mutations may be of interest based on the known functions of the affected genes, sequencing of GS populations did not support a determinant effect on HWSSL tolerance for every mutation. Namely, the mutations located in the 5′ UTR of *PBP1*, 3′ of *BCS1* and in *SGO1* and YNL058C seem to be linked in a single mutant that comprised a large proportion of the UV mutant population, but decreased in frequency throughout GS evolution. The verified tolerance-conferring effect of the *UBP7* mutation to which they were initially linked, and which gained in frequency over population evolution, suggests that these linked mutations were merely carried through to strain R57 from UV mutagenesis. The *TOF2* mutation gained in frequency within the GS-evolved strains at round 1 but diminished in later rounds, while the *DOP1* and *FIT3* 3′ UTR mutations were not highly represented in the GS populations and may be relatively specific to R57, which devalues these genes as potential tolerance-associated determinants. One exception is the mutation in Ste5p, which increased in frequency throughout GS, suggesting that it may play a role in the evolution of R57. However, since Ste5p is involved in mating, which is essential to the GS method used to generate R57, a strain with a modified mating behaviour may play a role in GS evolution but is likely not linked to stress tolerance.

## Conclusions

GS theory asserts that beneficial mutations accumulate within strains to rapidly evolve a trait of interest while simultaneously eliminating detrimental mutations. Our data suggest that during GS evolution, there is accumulation of complementary mutations in key cellular processes through recursive genetic recombination or by the accumulation of single mutations in crucial genes that confer a fitness advantage. Mutations that lead to a large fitness advantage, such as *UBP7*, may become highly represented in the initial mutant pool and lead to over-representation of non-productive mutations found in the same strain through genetic linkage. The decrease in the frequency of these non-productive mutations during GS evolution suggests that mutations of lesser or no impact on the trait of interest can be crossed out of final strains.

As a workflow, meiotic recombination-mediated GS of *S. cerevisiae*, combined with genome resequencing, population sequencing of mutation loci and RNA-seq transcriptional profiling, generated complementary results that provided novel insights into tolerance to a specific lignocellulosic hydrolysate, along with gene targets that can be used for strain engineering. The assortment of processes and genes involved in inhibitor tolerance could not have been rationally determined prior to this study. This study provides insights into the multiple biological processes that act in concert to establish tolerance to a multi-inhibitory substrate. Our strongest evidence supports determinant roles for Ubp7p and Art5p, related to ubiquitin-mediated proteolysis; stress response transcriptional repressor, Nrg1p; and NADPH-dependent glutamate dehydrogenase, Gdh1p. However, important roles in hydrolysate tolerance are supported for several of the mutations discovered in the GS-evolved strains and populations (*SSA1*, *ARO1*, *MAL11* and *GSH1*), and the potential phenotypic impact of each mutation has not been ruled out. Therefore, a subsequent study is ongoing in our lab to examine the effect of each mutation discovered in R57 and explore potential epistatic effects between the mutations reported here. As whole genome sequencing becomes ever more accessible, and as the biotechnology industry requires biocatalysts with increasingly complex traits, GS followed by analyses like those carried out in this study stands to have a rapid and profound effect on our understanding of complex multigenic traits.

## Materials and methods

### Strains and materials

The *S. cerevisiae* CEN.PK strains, supplied by EUROSCARF (Institute for Molecular Biosciences, Frankfurt, Germany), were used as the WT reference and progenitor strains for genome-shuffled mutant populations, including prototrophic diploid strain CEN.PK 122 and haploid strains CEN.PK 113-1A (*MAT***α**) and 113-7D (*MAT***a**). The haploid CEN.PK strains were used in a previous study to generate HWSSL-tolerant strain R57.

The HWSSL used for all experiments was provided by Tembec Inc. (Temiscaming, Quebec, Canada). HWSSL was adjusted to pH 5.5 with 10 M NaOH and contained, on average (*w*/*v*), 0.076% arabinose, 2% xylose, 0.16% galactose, 0.24% glucose, 0.43% mannose, 1% acetic acid, 0.18% furfural and 0.11% HMF.

### Sequencing of WT and R57 strains

Genomic DNA of WT haploid CEN.PK113-7D and the CEN.PK-derived R57 diploid yeast strains were isolated from overnight cultures grown in YPD using the DNeasy Blood & Tissue Kit (Qiagen, Toronto, Ontario, Canada). Library construction and sequencing were done at the Michael Smith Genome Sciences Centre using an Illumina 1G Genome Analyzer (Illumina, Inc., San Diego, CA, USA). Two lanes were sequenced for the WT strain and four for strain R57. All genome sequence data and RNA-seq read data from this publication have been submitted to the National Center for Biotechnology Information (NCBI) sequence read archive under BioProject # PRJNA231093.

### Sequencing alignment and mutation calling

Sequencing reads were aligned to the WT CEN.PK113-7D reference genome obtained from the NCBI (PRJNA52955) [77] and cross-referenced with read alignments obtained in our laboratory using our WT consensus sequence that was created using the S288c genome sequence as an alignment backbone. Alignments were performed both with Bowtie using standard parameters [78] and CLC Genomics Workbench version 5.1 with default parameters and ignoring non-specific matches. SNP and indel calling were both performed with Maq version 0.7.1 [79] and CLC Genomics Workbench for verification and visualization. To eliminate false positives in mutation calling, the DNA sequencing reads obtained from the WT were subjected to the same variation calling protocol as strain R57. Variations that were called when CEN.PK113-7D Illumina reads were aligned onto the CEN.PK113-7D consensus genome sequence and those that corresponded to variations called for R57 reads aligned onto the CEN.PK113-7D consensus sequence were discarded as false positives. False positive SNP calls were likely derived from sequence-specific miscall errors [80] or due to alignments in non-specific regions of the genome or to areas of low complexity [81]. The coverage requirement for SNP calling for WT reads was also lowered to ≤5-fold in order to ensure that SNPs that may result due to misalignment could be easily identified. These miscalls were edited out of the final mutation list by manual inspection and visualization with CLC Genomics Workbench. Maq SNP analysis was performed using the cns2snp command, and SNPs were called if the region upon which they were mapped returned a genome copy score = 1 and carried a Phred-like quality score ≥40. SNP analysis was corroborated with CLC Genomics Workbench SNP Detection function with a quality score of ≥40 for the central base and ≥30 for the surrounding bases. The threshold for variation at a specific base in the genome needed for SNP calling was lowered to ≥10% of reads, yielding an aberrant base call for the CEN.PK113-7D read alignment from ≥35% for R57 SNP calling in order to maintain stringency on positive variation calling. Therefore, if a SNP was called for more than 35% of the reads in R57 but was also called for 10% or more of the reads for the WT control, it was discarded as a false SNP call. Indel analysis was also performed with Maq using the Indelpe command and verified with CLC Genomics Workbench, both under standard parameters and compared to WT reads for control, as described above. Copy number variation was assessed using CNV-seq [82] to compare WT to R57 reads with a log2 threshold of 0.75, below the level used to detect reliable CNVs in CEN.PK [77]. The mutations identified were verified by Sanger sequencing and compared to the other Mat parental WT, CEN.PK113-1A, to ensure the mutations were not present in either of the WT parental haploid strains. Locations of mutations were assessed using the CEN.PK consensus sequence as a guide.

### Protein impact assessment of ORF-located mutations

Mutations in ORFs were examined by translating DNA sequences using ExPASy translate [83] and performing a BLAST comparison using the BLAST2Seq software program hosted by NCBI. Mutational impact assessment was done with the SIFT program [28,29] using recommended best practices [28]. Homologous proteins with <90% identity were chosen for comparison of the degree of conservation of the amino acid position in question for up to 100 homologous proteins. Amino acid substitutions were predicted as leading to a phenotype if the SIFT score was ≤0.05 and tolerated if the score is >0.05.

### Sequencing of R57 SNPs in GS heterogeneous populations

The following heterogeneous populations of cells were used to track R57 SNP frequency through GS evolution: WT diploid CEN.PK122, a pooled UV mutagenized population (three pools of CEN.PK113-1A and two pools CEN.PK113-7D UV mutants), along with cells from the population of the first, third and fifth rounds of recursive GS obtained from our previous study that generated R57 [8]. Cells from each population were selected from above the frontier of WT growth as observed by screening on HWSSL gradient agar plates (Figure 1B), yielding populations that were enriched for tolerance to HWSSL, as described [8]. A sample of 10^9^ cells, suspended in phosphate-buffered saline (PBS), from each population was spread onto a single lane of the gradient plate and incubated for 6 days at 30°C. Each plate contained two lanes spread with CEN.PK 122 cells for comparison to each individual GS population, in duplicate. Each plate was screened in biological duplicates.

Cells that grew to higher HWSSL concentrations than the WT on the gradient plates were scraped, suspended in PBS and adjusted to approximately 4 × 10^8^ cells/mL using a haemocytometer. For each population, DNA from approximately 4 × 10^7^ cells was extracted using a DNeasy Blood & Tissue Kit (Qiagen, Toronto, Ontario, Canada). Five microlitres of each genomic DNA preparation was used as template for PCR with primers specific for each of the 20 SNP regions, located at approximately 50 bp from either end of the SNP. The primers were designed with sequencing adapter attachment for use with the Ion Torrent Personal Genome Machine (Life Technologies, Carlsbad, CA, USA) according to the manufacturer’s instructions (Additional file 2: Table S1). All PCR products were gel purified and quantified in triplicate using the Promega Quantifluor dsDNA system (Promega Corporation, Madison, WI, USA). The PCR products were diluted to 16 pmol, and 20-μL samples from each reaction were pooled to make four pools (one UV mutagenized and three GS pools). Pools of PCR products were sequenced using a 316 chip with the 200-bp kit (following the Ion Torrent protocols).

### Sequencing SNPs from isolates of the round 5 GS heterogeneous population

Twenty isolated colonies picked from the growth frontier of a HWSSL gradient plate of the round 5 GS population were streak purified on 50% (*v*/*v*) HWSSL and 2% agar (*w*/*v*) Petri plates. Colonies isolated from the plates were grown overnight in 5 mL YPD broth at 30°C, and DNA was extracted with a DNeasy Blood & Tissue Kit for use as a template in the PCR amplification of the mutated gene region. Primers used for amplification are described in Additional file 2: Table S2, and PCR products were sequenced using the Sanger method.

### Transcriptome analysis

WT diploid strain CEN.PK122 and HWSSL-tolerant R57 were used in RNA-seq experiments. The WT and R57 strains were grown in 50 mL synthetic defined (SD) medium (yeast nitrogen base without amino acids 0.17% *w*/*v*, ammonium sulphate 0.5% *w*/*v*, glucose 2% *w*/*v*) overnight at 30°C under semi-fermentative conditions (sealed 125-mL flasks shaken at 100 rpm) to early stationary phase (Additional file 3: Figure S4). Cultures were normalized to an OD_600 nm_ of 3, and two independently grown cell samples of these cultures were used for RNA extraction. For each sample, cells from 5 mL of culture were harvested by centrifugation at 1,800 × *g* and 4°C and frozen in liquid N_2_ until RNA isolation. RNA extracts were prepared using the RNeasy Plant Mini Kit (Qiagen) according to manufacturer’s specifications for use with yeast, in which frozen cells were suspended in lysis buffer and disrupted with a mini bead beater (Precellys 24, Bertin Technologies, Montigny-le-Bretonneux, France) at 4°C. Prior to sequencing, RNA quality was confirmed using an Agilent 2100 Bioanalyzer (Agilent Technologies, Santa Clara, CA, USA).

RNA sequencing was performed at the McGill/Genome Quebec Innovation Centre in duplicate on the Illumina Genome Analyzer *IIx* and Illumina HiSeq 2000 for the WT *vs*. R57. DNA libraries were subjected to 36 or 50 cycles of sequencing on the Illumina Genome Analyzer *IIx* and Illumina HiSeq 2000, respectively.

RNA-seq differential transcription analysis and statistical comparisons were performed with CLC Genomics Workbench version 5.1. The cDNA sequence reads from RNA-seq were trimmed to remove Illumina sequencing adaptors as well as unreliable read ends, and alignments were performed using the CEN.PK113-7D genome sequence and associated GTF file [77] as the backbone for alignment mapping and quantitation. Significance values for differential expression were computed using Baggerly’s test [84]. The samples were then FDR-corrected in order to eliminate non-productive leads from the expression results. Gene transcripts showing differential expression with a corrected *P* value of <0.05 and a >2-fold increase were used for functional clustering and enrichment mapping of differentially expressed genes.

Functional annotation clustering was executed with DAVID Bioinformatics Resources 6.7 [85]. Clusters of up or down expressed genes with gene ontology (GO) term enrichment scores of ≥1.3 (equivalent to a non-log scale value of 0.05) are reported, unless stated otherwise. Enrichment maps of ontology categories from clustering were generated with the Enrichment Map 1.2 software plug-in for Cytoscape 2.8 [86,87]. All functional annotations presented were derived from SGD [88] or the DAVID server unless otherwise referenced. Transcription factor binding analysis was done through the YEASTRACT database [89-91].

### Reconstitution of the *UBP7* mutation in WT

To determine if the *UBP7* mutation was contributing to the HWSSL tolerance phenotype of R57 as predicted, the mutation was introduced into WT and the resulting strain was tested for growth on HWSSL. The WT allele was replaced in CEN.PK113-7D via homologous recombination of a DNA cassette containing the mutated *UBP7* sequence flanked by a kanamycin resistance marker. Sanger sequencing of the PCR-amplified region was used to confirm that the transformants harboured the mutation. Homozygous diploid strains of the WT and *UBP7* mutant were created by mating type switching using the YCp50::HO plasmid [92] and mating haploid strains of opposite mating type. The *UBP7* homozygous diploid mutant and WT diploid strains along with R57 were tested for their tolerance to HWSSL in parallel, as previously described [8].

## Additional files

Additional file 1: Data S1. A summary mapping report for CEN.PK 113-7D. Additional file 2:
**Supplemental methods.** A document on assessing functional interaction of mutations. (DOCX 31 kb) Additional file 3:
**Supplemental figures.** **Figure S1.** Interaction map of genes affected by mutation in R57. **Figure S2.** Ontology categories associated with R57 genes affected by mutation. **Figure S3.** Differentially expressed genes comprising enrichment clusters based on biological function between the WT and R57. **Figure S4.** Cell growth and glucose consumption of WT and R57 mutant strains. Additional file 4: Data S2. RNA-seq results for differentially expressed genes between WT *vs* R57 without HWSSL exposure.

## Abbreviations

CNV: copy number variation
GS: genome shuffling
HMF: 5-(hydroxymethyl)-2-furaldehyde
HWSSL: hardwood spent sulphite liquor
indel: insertion/deletion
NBD: nucleotide-binding domain
UBL: ubiquitin-like
