# Supplementary material for: Deconstructing the genetic basis of spent sulphite liquor tolerance using deep sequencing of genome-shuffled yeast
Source: Biotechnol Biofuels. 2015 Mar 31;8:53. doi: 10.1186/s13068-015-0241-z (PMC4393574; doi:10.1186/s13068-015-0241-z)
Supplement: Additional file 2: — Supplemental methods. A document on assessing functional interaction of mutations. (DOCX 31 kb) [file 13068_2015_241_MOESM2_ESM.docx]

### Supplemental methods

### Assessing functional interaction of mutations

Analysis of potential functional interaction was carried out on the 16 genes non-ambiguously affected by mutations through analysis with the BiNGO version 2.44 [1] software plugin for the Cytoscape 2.8 network analysis software program [2-4]. Mutant genes were annotated with the complete BiNGO Gene Ontology (GO) file to visualize the full range of known functional relationships between the genes of interest (Supplemental Fig. 6) or the GOSlim yeast annotation file for broader visualization of affected functional categories. Protein-protein, genetic, co-expression, co-localization, predicted functional relationship, and shared protein domain interactions between products of the genes affected by mutation were explored using the GeneMANIA version 3.2 [5, 6] software plugin for Cytoscape 2.8. The 20 most related genes as determined by GeneMANIA were included within the interaction network to explore non-direct interrelationships. Weighting for the GeneMANIA generated interaction map (Supplemental Fig. 7) was based on GO molecular functional relationships, while evidence scoring was based on assigned relevance of the sources used to generate interactivity by the software. All annotations of genes are based on the *Saccharomyces* Genome Database (SGD) unless otherwise referenced.

### Supplemental References

1. Maere S, Heymans K, Kuiper M: **BiNGO: a Cytoscape plugin to assess overrepresentation of gene ontology categories in biological networks.** *Bioinformatics* 2005, **21:**3448-3449.

2. Smoot ME, Ono K, Ruscheinski J, Wang PL, Ideker T: **Cytoscape 2.8: new features for data integration and network visualization.** *Bioinformatics* 2011, **27:**431-432.

3. Shannon P, Markiel A, Ozier O, Baliga NS, Wang JT, Ramage D, Amin N, Schwikowski B, Ideker T: **Cytoscape: a software environment for integrated models of biomolecular interaction networks.** *Genome Res* 2003, **13:**2498-2504.

4. Cline MS, Smoot M, Cerami E, Kuchinsky A, Landys N, Workman C, Christmas R, Avila-Campilo I, Creech M, Gross B, et al: **Integration of biological networks and gene expression data using Cytoscape.** *Nat Protoc* 2007, **2:**2366-2382.

5. Montojo J, Zuberi K, Rodriguez H, Kazi F, Wright G, Donaldson SL, Morris Q, Bader GD: **GeneMANIA Cytoscape plugin: fast gene function predictions on the desktop.** *Bioinformatics* 2010, **26:**2927-2928.

6. Warde-Farley D, Donaldson SL, Comes O, Zuberi K, Badrawi R, Chao P, Franz M, Grouios C, Kazi F, Lopes CT, et al: **The GeneMANIA prediction server: biological network integration for gene prioritization and predicting gene function.** *Nucleic Acids Res* 2010, **38:**W214-220.

**Supplemental Table S1.** Primers used for PGM sequencing. Primers used to amplify mutated regions and add the adapter regions and key sequence for Ion Torrent sequencing. Adapter region P1 is depicted in bold in the forward primers while adapter region A is depicted in bold in the reverse primers. The key sequence is italicized in the reverse primers.

| **Primer Name** | **Primer** |
| --- | --- |
| SSA1_F_PGM | **CCTCTCTATGGGCAGTCGGTGAT**GCATCACCAATCAATCTTTCAGTGTC |
| SSA1_R_PGM | **CCATCTCATCCCTGCGTGTCTCCGAC***TCAG*CGTGTGTTGCTCACTTTGCTAATG |
| TOF2_F_PGM | **CCTCTCTATGGGCAGTCGGTGAT**CAGCTGGTCCATGACTTGTTTTG |
| TOF2_R_PGM | **CCATCTCATCCCTGCGTGTCTCCGAC***TCAG*CTTCCATGTCACTTGAATCTTCATTG |
| UBP7_F_PGM | **CCTCTCTATGGGCAGTCGGTGAT**CCATGAAGTTGAGTAAACTTGGTAGG |
| UBP7_R_PGM | **CCATCTCATCCCTGCGTGTCTCCGAC***TCAG*GGCAATCCCATGCATTTTCACC |
| YNL058c_F_PGM | **CCTCTCTATGGGCAGTCGGTGAT**GGAAGGTCTCTTCTGACCAGC |
| YNL058c_R_PGM | **CCATCTCATCCCTGCGTGTCTCCGAC***TCAG*CTAGACTCATACCTTGTCAAAAGTTC |
| MAL11a_F_PGM | **CCTCTCTATGGGCAGTCGGTGAT**CCTTCCATAACCAGGGTAGTAG |
| MAL11a_R_PGM | **CCATCTCATCCCTGCGTGTCTCCGAC***TCAG*GCTAACAGCGAGGAAAAAAGCATG |
| MAL11b_F_PGM | **CCTCTCTATGGGCAGTCGGTGAT**CCATATAAGTCGTGATTTGCAAACC |
| MAL11b_R_PGM | **CCATCTCATCCCTGCGTGTCTCCGAC***TCAG*GGAGGGTTCTTACGAAATTACTTCC |
| GDH1a_F_PGM | **CCTCTCTATGGGCAGTCGGTGAT**CCTCTTTGGAAGACTCTACTCTTTTC |
| GDH1a_R_PGM | **CCATCTCATCCCTGCGTGTCTCCGAC***TCAG*CCCAGGTGACTCTGAATTGTATG |
| GDH1b_F_PGM | **CCTCTCTATGGGCAGTCGGTGAT**GTCAGAGCCAGAATTTCAACAAGC |
| GDH1b_R_PGM | **CCATCTCATCCCTGCGTGTCTCCGAC***TCAG*GCAAAACCTTTCTGTATTCTGGGTG |
| GSH1_F_PGM | **CCTCTCTATGGGCAGTCGGTGAT**GTATATTTTCGATACTCTAAACCACCC |
| GSH1_R_PGM | **CCATCTCATCCCTGCGTGTCTCCGAC***TCAG*GCTGGAGTAGTTGGATCTTTCC |
| DOP1_F_PGM | **CCTCTCTATGGGCAGTCGGTGAT**GCTCGACATTAGCACGAAACTTC |
| DOP1_R_PGM | **CCATCTCATCCCTGCGTGTCTCCGAC***TCAG*GGATCGACAAAAAATGTCCTTACCAC |
| SGO1_F_PGM | **CCTCTCTATGGGCAGTCGGTGAT**GCATGAATCAAGTTTTAACAAGGACG |
| SGO1_R_PGM | **CCATCTCATCCCTGCGTGTCTCCGAC***TCAG*CGGTTTCGTCTTCAGGTTCTAAAC |
| BCS1_F_PGM | **CCTCTCTATGGGCAGTCGGTGAT**CGACATGGTAGATTGAGGGC |
| BCS1_R_PGM | **CCATCTCATCCCTGCGTGTCTCCGAC***TCAG*GTAAAGATTTCCACTCTTCTATATTTGC |
| RPB11_F_PGM | **CCTCTCTATGGGCAGTCGGTGAT**CGGATAGCAGCATTATCCATGAC |
| RPB11_R_PGM | **CCATCTCATCCCTGCGTGTCTCCGAC***TCAG*GAATTTACAGACTACTGGAGAGGG |
| FIT3_F_PGM | **CCTCTCTATGGGCAGTCGGTGAT**GTGTGTTATTAAAATTTTTTTATTCTAACATAACTTCG |
| FIT3_R_PGM | **CCATCTCATCCCTGCGTGTCTCCGAC***TCAG*CCGTCATGTTATTGTAAATGATATGTG |
| ARO1_F_PGM | **CCTCTCTATGGGCAGTCGGTGAT**CCCTGCTGATCAACAGAAAGTTG |
| ARO1_R_PGM | **CCATCTCATCCCTGCGTGTCTCCGAC***TCAG*GATTTTACATTGACCTTCACCGAGG |
| ART5_F_PGM | **CCTCTCTATGGGCAGTCGGTGAT**GCAGTACAAGCAACCAAGATATGG |
| ART5_R_PGM | **CCATCTCATCCCTGCGTGTCTCCGAC***TCAG*CTTCGAATTATTTTGTTGAAAACAGGG |
| NRG1_F_PGM | **CCTCTCTATGGGCAGTCGGTGAT**GCAGTCTTATTTAATTTGTGTTTTAGTTCATTC |
| NRG1_R_PGM | **CCATCTCATCCCTGCGTGTCTCCGAC***TCAG*GGAAACGTTGAAATAAGCCCGG |
| PBP1_F_PGM | **CCTCTCTATGGGCAGTCGGTGAT**GGAAAGAACAAAAAGAAAGATAGAAGAAAAC |
| PBP1_R_PGM | **CCATCTCATCCCTGCGTGTCTCCGAC***TCAG*CGTTTTTGTAAAGCAGTTCTTAAATCG |
| STE5_F_PGM | **CCTCTCTATGGGCAGTCGGTGAT**GGTCGCTCCATTTGGCTATC |
| STE5_R_PGM | **CCATCTCATCCCTGCGTGTCTCCGAC***TCAG*CCCTTTCTTCTGTTAGAAATAGGC |

**Supplemental Table S2.** List of primers used to integrate the mutation found in *UBP7* into the chromosome**.**

| **Primer names** | **Primer sequence** |
| --- | --- |
| UBP7mut_1 | CCATGAAGTTGAGTAAACTTGGTAGGTCTACTGAGAAAAGAGTTAAGTTAGAGG |
| UBP7mut_2 | ACGAAGTTATATTAAGGGTTGTCGACCTGCAGCGTACGAAGCTTCAGCTGGGTGACAAA GATAACATTCACAAGAG |
| UBP7mut_3 | CTCTTGTGAATGTATCTTTGTACCCCAGCTGAAGCTTCGTACGCTG |
| UBP7mut_4 | GGAACACTGCCAGCGCATC |
| UBP7mut_5 | GATGCGCTGGCAGTGTTCC |
| UBP7mut_6 | CATCAAGACGTTTGGTGTCTAAATCGGCCGCATAGGCCACTAG |
