# Supplementary material for: Deconstructing the genetic basis of spent sulphite liquor tolerance using deep sequencing of genome-shuffled yeast
Source: Biotechnol Biofuels. 2015 Mar 31;8:53. doi: 10.1186/s13068-015-0241-z (PMC4393574; doi:10.1186/s13068-015-0241-z)
Supplement: Additional file 3: — Supplemental figures. Figure S1. Interaction map of genes affected by mutation in R57. Figure S2. Ontology categories associated with R57 genes affected by mutation. Figure S3. Differentially expressed genes comprising enrichment clusters based on biological function between the WT and R57. Figure S4. Cell growth and glucose consumption of WT and R57 mutant strains. [file 13068_2015_241_MOESM3_ESM.pdf]

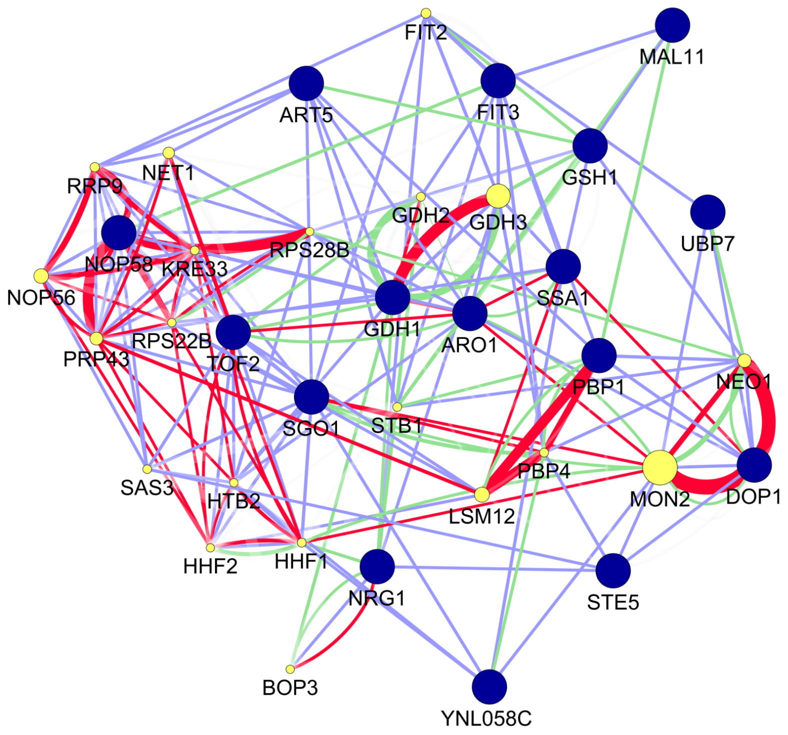

Supplemental Figure S1. Interaction map of genes affected by mutation in R57. Interaction maps were generated through GeneMANIA, drawing from extensive source annotations built into the software. Blue nodes are the genes affected by mutation in R57 (uniform size), while the yellow nodes are the top 20 interacting genes as determined by GeneMANIA (larger size equals a higher degree of interaction within the network). The edges connecting the nodes represent protein-protein (red lines), genetic (green lines) and co-expression (light-blue lines) interactions. Thicker lines represent a higher confidence of interaction.

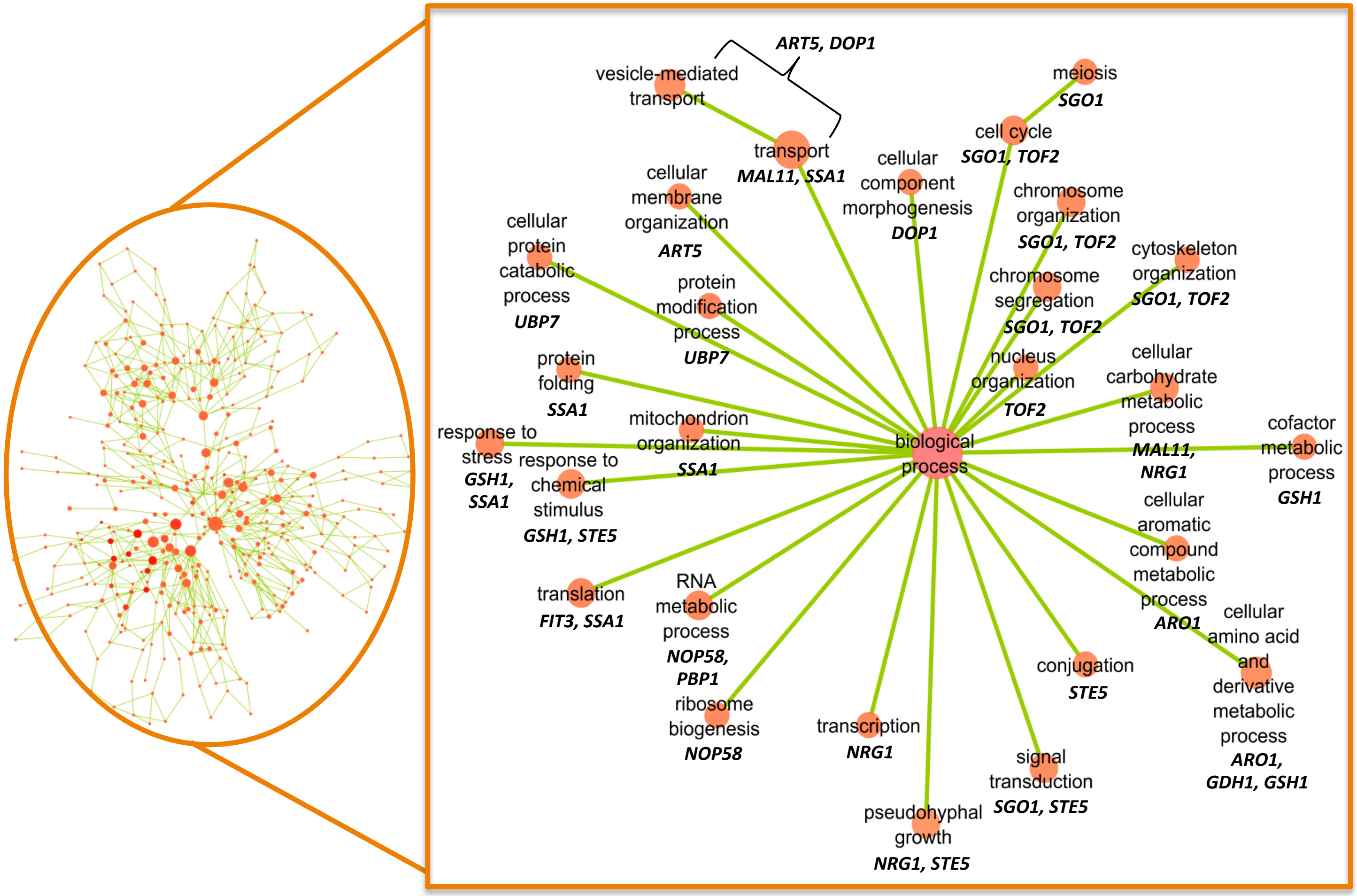

Supplemental Figure S2. Ontology categories associated with R57 genes affected by mutation. Using the full gene ontology set for yeast, a vast interaction network can be generated of biological processes potentially affected by mutation (left) and summarized using the GOSlim Yeast ontology categories (right). Genes affected by mutation (bold, Table 1) are listed under their associated ontology category (orange node).

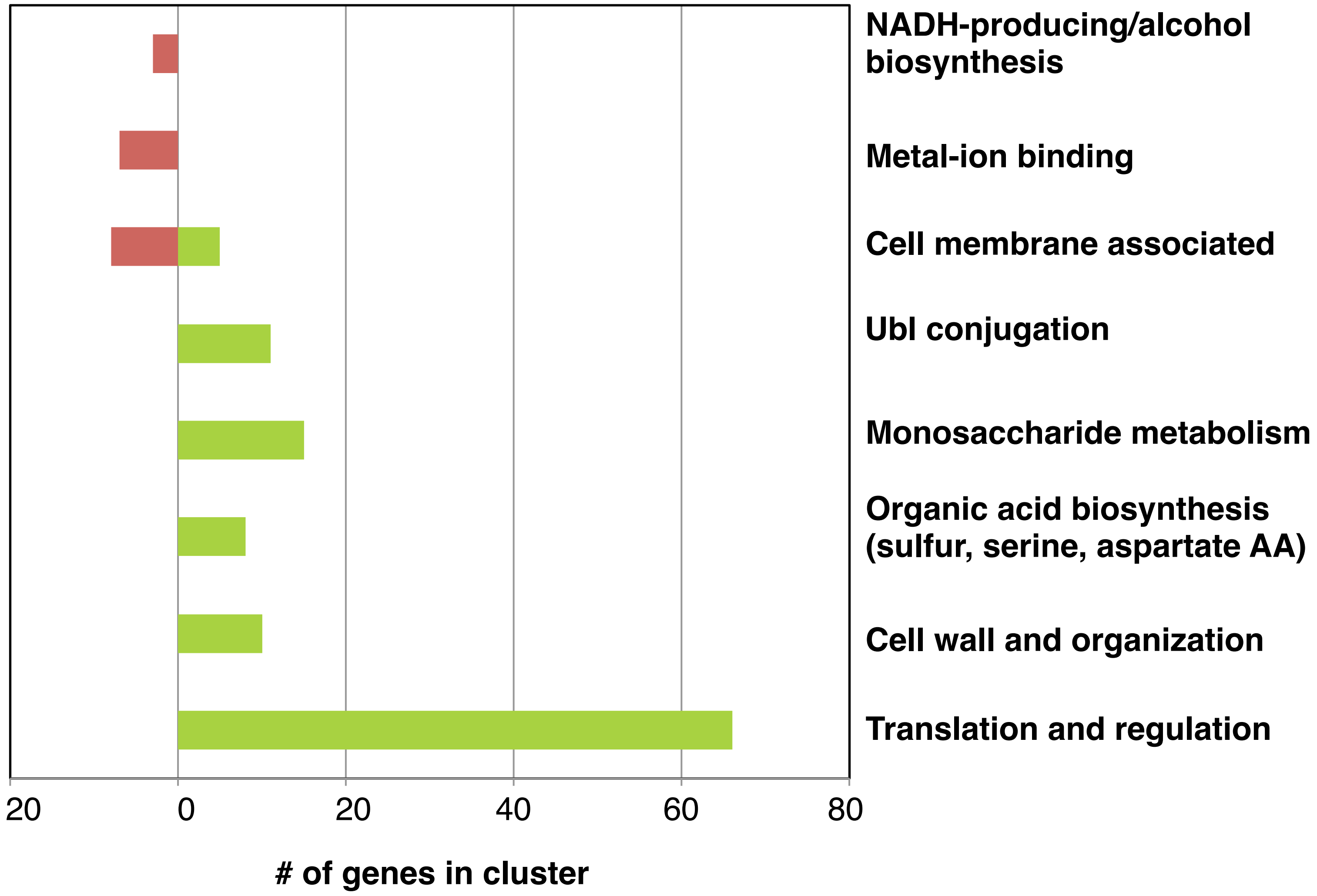

Supplemental Figure S3. Differentially expressed genes comprising enrichment clusters based on biological function between the WT and R57. The number of upregulated (> 2 fold, green) or downregulated genes (> 2-fold, red) are depicted based on associated GO term enrichment clustering with scores > 1.3.

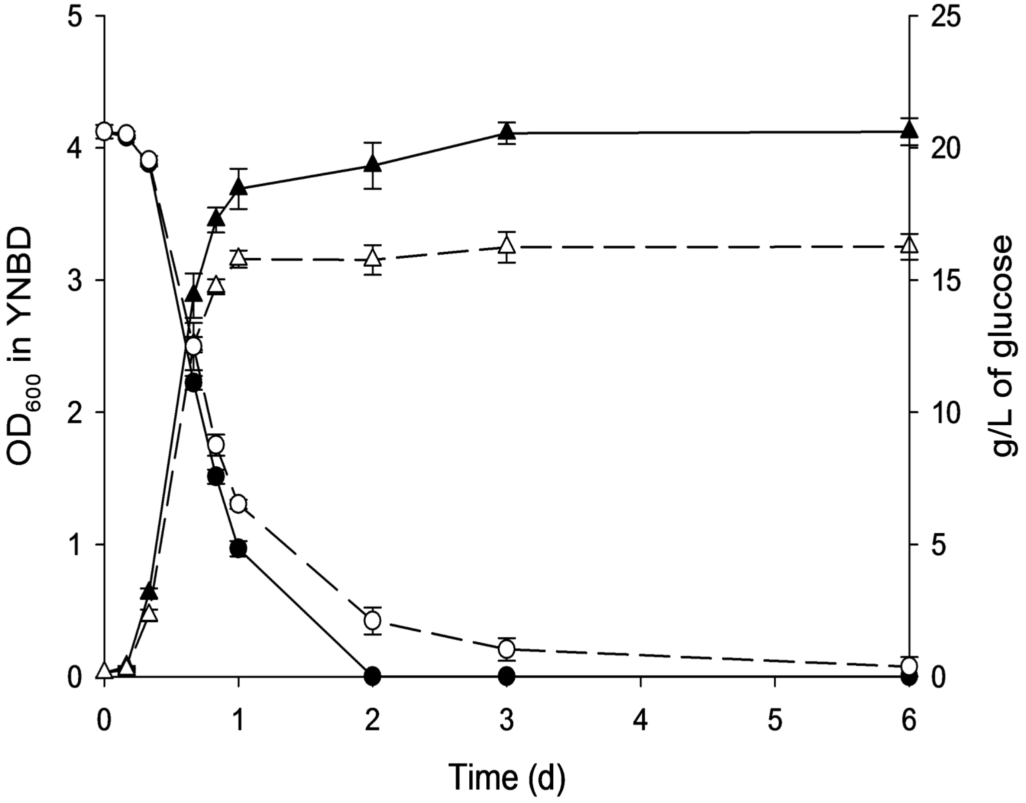

Supplemental Figure S4. Cell growth and glucose consumption of WT and R57 mutant strains. Overnight SD cultures of WT (solid symbols) and R57 (open symbols) were inoculated into 50 mL of SD medium with an initial OD600 of 0.03 (0 h) and then cells were cultured at 200 rpm at 30 °C in 250-mL flasks. Cell growth (left y-axis, triangles) was monitored by measuring OD600 at 4, 8, 16, 20, 24, 48, 72 and 144 hours. Glucose (right y-axis, circles), concentration in the medium was determined by HPLC.
